# Somatic Point Mutation Calling in Low Cellularity Tumors

**DOI:** 10.1371/journal.pone.0074380

**Published:** 2013-11-08

**Authors:** Karin S. Kassahn, Oliver Holmes, Katia Nones, Ann-Marie Patch, David K. Miller, Angelika N. Christ, Ivon Harliwong, Timothy J. Bruxner, Qinying Xu, Matthew Anderson, Scott Wood, Conrad Leonard, Darrin Taylor, Felicity Newell, Sarah Song, Senel Idrisoglu, Craig Nourse, Ehsan Nourbakhsh, Suzanne Manning, Shivangi Wani, Anita Steptoe, Marina Pajic, Mark J. Cowley, Mark Pinese, David K. Chang, Anthony J. Gill, Amber L. Johns, Jianmin Wu, Peter J. Wilson, Lynn Fink, Andrew V. Biankin, Nicola Waddell, Sean M. Grimmond, John V. Pearson

**Affiliations:** 1 Queensland Centre for Medical Genomics, Institute for Molecular Bioscience, The University of Queensland, Brisbane, Queensland, Australia; 2 The Kinghorn Cancer Centre, and the Cancer Research Program, Garvan Institute of Medical Research, Sydney, New South Wales, Australia; 3 Department of Surgery, Bankstown Hospital, Sydney, New South Wales, Australia; 4 South Western Sydney Clinical School, University of New South Wales, Liverpool, New South Wales, Australia; 5 University of Sydney, Sydney, New South Wales, Australia; Georgia Institute of Technology, United States of America

## Abstract

Somatic mutation calling from next-generation sequencing data remains a challenge due to the difficulties of distinguishing true somatic events from artifacts arising from PCR, sequencing errors or mis-mapping. Tumor cellularity or purity, sub-clonality and copy number changes also confound the identification of true somatic events against a background of germline variants. We have developed a heuristic strategy and software (http://www.qcmg.org/bioinformatics/qsnp/) for somatic mutation calling in samples with low tumor content and we show the superior sensitivity and precision of our approach using a previously sequenced cell line, a series of tumor/normal admixtures, and 3,253 putative somatic SNVs verified on an orthogonal platform.

## Introduction

The declining cost of next-generation sequencing is enabling an increasing number of tumor sequencing studies [1]–[3], providing new insights into the mutations driving tumorigenesis. These large-scale efforts are redefining the role of known oncogenes and tumor suppressor genes, identifying new candidate driver genes and providing insights into the mutational mechanisms at play in different tumor types [4], [5]. Accurate somatic mutation calling is paramount in these studies.

Despite this growing demand for accurate somatic mutation calls in cancer studies, mutation calling from next-generation sequencing data remains challenging. Early cycle PCR-induced errors, polymerase slippage [6] and the mis-mapping of reads due to homology to multiple genomic regions are some of the most common sources of false positive calls. Inadequate sequence depth in the matched normal sample can also result in germline variants being incorrectly identified as somatic mutations (false positives). Finally, tumor heterogeneity and purity further confound accurate somatic mutation calling as increased tumor heterogeneity and decreased purity result in lower mutant allele ratios that can make it difficult to distinguish true mutations from background (false negative error). In solid tumors, purity varies widely with some tumor samples having less than 10% tumor content. Many low purity tumor samples have been excluded from somatic mutation analysis to date due to the analytical challenges associated with accurately calling mutations in these samples and the expected high false negative rate. To keep the sensitivity of the analysis at desired levels, there is a risk of calling an increasing number of false positives.

Several software programs have been developed for variant and somatic mutation calling, including GATK [7], Strelka [8], diBayes (Applied Biosystems BioScope™ software), SomaticSniper [9], VarScan 2 [10] and SNVMix [11]. For cancer genome analysis and to identify somatic events, a tumor sample is compared to its matched normal sample. Current software tools differ in important ways by either performing single or joint sample analysis of the tumor/matched normal sample pair, and by either using Bayesian or heuristic approaches (Table 1). GATK was initially developed in the context of the 1000 Genomes Project [12] to enable variant discovery and genotyping from next-generation sequencing data. GATK performs single sample analysis only. A tumor and matched normal sample pair are thus genotyped independently and somatic events are determined by subtracting calls in the normal from those in the tumor sample. In contrast, Strelka, SomaticSniper and VarScan 2 perform joint sample analysis of a tumor/normal pair and either model tumor as a mixture of normal sample with somatic variation (Strelka), calculate joint diploid genotype likelihoods using the MAQ genotype model (SomaticSniper) or compare read count distributions between the two samples using Fisher's exact test (VarScan2). Importantly, due to the different statistical models employed, current somatic mutation callers differ in the number of somatic mutation calls and in their overlap. In addition, many somatic mutation callers use a series of post-call filtering steps that further affect the number and type of final mutation calls. Some of these tools also allow analysis of small indels, germline variants and copy number variations (Table 1).

**Table 1:** Variant calling software tools.

Software	Tumor normal joint analysis	Output germline variants	Indels	Statistical method	Reference
qSNP	X	X		empirically determined set of heuristics optimized for sensitivity in low purity tumors	present study
GATK		n/a	X	Bayesian model for genotype likelihood, can take into account multiple samples for calibration	[7]
Strelka	X		X	Bayesian model of tumor as a mixture of normal sample with somatic variation	[8]
SomaticSniper	X	X		Bayesian comparison of genotype likelihoods based on MAQ genotype model	[9]
diBayes		n/a		Bayesian model for presence of non-reference allele (color-space data)	Applied Biosystems BioScope™
VarScan2	X	X	X	heuristics to determine genotypes and Fisher's exact test to examine read count differences, also outputs CNV regions for exome data	[10]
SNVMix		n/a		probabilistic binomial mixture model accounting for tumor ploidy and purity	[11]

There have not been, however, any detailed investigations of the effects of reduced tumor cellularity or purity on the accuracy and sensitivity of somatic mutation calling, although a recent software, MuTect, has been designed especially with subclonal mutations in mind [13]. A number of factors compromise somatic mutation calling in low purity tumors. As sequence coverage and tumor purity decrease, the effects of allele sampling confound the accurate assessment of allele distributions and thus compromise statistical models for determining potential variant sites of interest. Secondly, depending on the statistic models used, low frequency mutations may or may not trigger a variant call resulting in differences in the number and type of mutations called between different callers. Our interests in pancreatic adenocarcinomas where over 70% of tumors are of less than 40% purity due to desmoplastic stroma and despite enrichment by histology-guided macrodissection [14] have motivated us to determine optimal strategies for somatic point mutation calling in these tumors. To this end, several mutation calling strategies were tested and extensive verification was performed, in which true positive and false positive mutation calls were inspected to identify common error sources. A heuristics-based single nucleotide variant caller, qSNP, was then implemented using these empirically determined features. Its performance was directly assessed in samples of varying purity that were generated by mixing a tumor cell line and its matched normal sample at varying proportions and sequencing each mixture. The decay in sensitivity as purity decreased in these mixtures was assessed and the performance of our caller was compared to that of two others. Finally, its performance was benchmarked against the COLO-829 cell line, previously sequenced and analyzed by Pleasance *et al.* [4].

## Results

Our somatic mutation calling strategy has been designed to maximize sensitivity in light of low tumor purity. Iterative rounds of verification using benchtop amplicon-based sequencing were performed to develop and refine post-processing checks to control the false discovery rate. The following considerations informed the design of our mutation calling strategy and its software implementation, qSNP.

### Joint analysis of the tumor and matched normal sample

qSNP considers sequence data in Binary Sequence Alignment/Map (BAM) format [15] from both tumor and matched normal samples jointly. Classification into germline and somatic calls follows a number of simple rules that were designed to accommodate for the expected low mutant allele ratio in low purity tumors (Table 2).

**Table 2:** Classification of germline and somatic events.

Normal genotype	Tumor genotype	Details*	Classification
Hom	Het	Variant is reference allele; G/G>A/G	Germline^1^
Hom	Het	Variant novel; A/A>A/G	Somatic^1^
Het	Hom	Tumor allele same; A/G>G/G	Germline^2^
Het	Hom	Tumor allele different; A/G>T/T	Somatic
Hom	Hom	Same; G/G>G/G	Germline
Hom	Hom	Different; A/A>G/G	Somatic
Het	Het	Same; A/G>A/G	Germline
Het	Het	Different; A/G>T/G	Somatic

* All examples assume ‘A’ as the reference allele, ‘G’ as the variant, and ‘Hom’ and ‘Het’ denote homozygous and heterozygous respectively.

1 check coverage in normal to exclude under-calling.

2 could indicate LOH in tumor.

### Maximize sensitivity of mutation calling

qSNP currently triggers a variant call if a minimum of 3 reads of the same, non-reference allele are found. We found that this minimum evidence requirement ensures that a variant call is triggered even in regions where Poisson sampling of alleles may have confounded the observed allele distributions. As sequence depth increases, so does the minimum read requirement. At coverage over 20× a minimum of 4 mutant reads are required and above 50× a minimum of 5% of mutant reads or a minimum of 2.5% mutant reads if reads are on both strands. In addition, the base qualities of the variant reads must be at least 10% of the sum of base qualities at the position or at least 5% of the sum of base qualities if reads are found on both strands and coverage is over 50×. To determine whether the position is homozygous or heterozygous, the two most common alleles are determined. If both alleles match the evidence criteria above, the position is considered heterozygous, and if not, homozygous.

### Post-processing checks to control the false discovery rate

Various factors influence the confidence in a somatic mutation call, including sequence depth in tumor and matched normal, base qualities of alleles, evidence for variant in matched normal sample, number of mutant reads, and mutant allele ratio. The statistical frameworks to encompass all of these factors into a single model and metric are still being developed. Some single-sample SNP callers give a p-value that purely reflects the likelihood for the presence of a non-reference allele. Furthermore, most mutation calling software is used in combination with a series of post-calling filtering steps to remove likely false positives. This practice means that the original p-values calculated by the mutation caller are overridden by these further checks that ultimately decide whether or not a mutation is considered high confidence. For low purity tumors, Poisson sampling of alleles can confound estimates of their true frequencies, further compromising the calculation of accurate p-values or resulting in positions not exceeding a likelihood threshold.

For these reasons we have not made an attempt to estimate a p-value upfront but instead use flags to indicate that a putative somatic mutation call does not meet certain quality criteria or evidence thresholds (Table 3). For example, putative somatic positions are checked for the presence of the variant in the matched normal BAM. If a position has evidence in the normal, the call is annotated as such. Somatic positions are further checked for being a germline variant in another patient as this can indicate under-sampling of alleles in the matched normal. For this check, we use an in-house database of germline variants and qSNP can be set up to output high quality germline calls to this database with each iteration of qSNP. Positions that pass all checks are considered to be of highest confidence and we expect these to be true somatic events. They are annotated as PASS in the qSNP output. Positions where the normal sample lacks adequate sequencing coverage are potentially false positive somatic calls and may return germline in verification. These are annotated as COVN12 in qSNP output. All remaining somatic mutations such as those where there is evidence of the variant also in the normal sample or where only few mutant reads support the variant call are considered lowest confidence and are expected to include many false positives. These calls are annotated as outlined in Table 3.

**Table 3:** Post-processing checks performed by qSNP.

Annotation	Variant type	Description
PASS	Somatic, Germline	(Passed all post-processing checks) AND (min 5 mutant reads) AND (min 4 novel starts not considering read pair)
COVN12	Somatic	Less than 12 reads coverage in matched normal sample
COVN8	Germline	Less than 8 reads coverage in matched normal sample
SAN3	Germline	Less than 3 reads of same allele in normal
COVT8	Germline	Less than 8 reads coverage in tumor
SAT3	Germline	Less than 3 reads of same allele in tumor
GERM	Somatic	Mutation is a germline variant in another patient
MIN	Somatic	Mutation also found in pileup of normal BAM
MIUN	Somatic	Mutation also found in pileup of unfiltered normal BAM
NNS	Somatic, Germline	Less than 4 novel starts not considering read pair
MR	Somatic, Germline	Less than 5 variant reads
MER	Somatic	Mutation same as reference
SBIAS	Somatic	Strand bias (Illumina only)

### Output mutation calls in.vcf and DCC formats

Output in Variant Call Format (VCF) [16] was required as VCF is becoming the standard format for mutation reporting and annotation and allows integration with an ever-expanding set of VCF tools. To enable easy integration with the International Cancer Genome Consortium (ICGC) Data Coordination Centre (DCC), output in DCC format was also implemented.

### Fast, easy to run and operating-system independent

Given the continuously increasing throughput of next-generation sequencing platforms, qSNP needed to be efficient in its use of compute resources. To achieve this, qSNP is implemented in JAVA using the Picard library (version 1.62). qSNP is driven by a single plain-text configuration file in the “Windows INI-file” style and takes as its primary inputs, a pair of tumor and normal BAM files that have been duplicate-marked and coordinate-sorted. qSNP implements a fast and flexible read-filtering system and if filters such as minimum mapping quality or alignment length are specified, qSNP will filter out failing reads prior to analysis. qSNP creates a pileup of bases in tumor and normal to look for evidence of a variant. qSNP has been specifically designed to make use of a compute cluster. It is thus multi-threaded, requiring 5 cores and 20 GB of memory to run efficiently.

### Tuning using verification data

To identify common error sources and to refine qSNP, extensive verification of 3,253 putative somatic mutation calls was performed across 65 tumors of 6 to 83% purity (mean 38% purity), including 60 tumors reported in Biankin *et al.* [14] (Table 4, Table S1). In total, 717 mutations were confirmed as true somatic events, of which 704 had been classified as PASS by qSNP (Table 4). Miscalled somatic mutations were most commonly associated with one of three features: position in regions of sequence homology, support only by non-independent reads or support by low evidence. By designing strategies to eliminate false positives associated with these common error sources, we were able to maintain an accuracy of 57% at a sensitivity of 98% across these tumors of mean purity of 38% (Table 4). This sensitivity is likely an overestimate of the true sensitivity as only known, verified mutations called by qSNP at any evidence threshold were chosen for verification; it is possible that there were additional somatic events that were never called. Nevertheless, our strategy is successful in retaining the vast majority of *known* true positive events (98%), while eliminating false positive calls associated with common error sources.

**Table 4:** Details of verification using amplicon-based sequencing on the Ion Torrent.

Verification across65primary pancreatic adenocarcinomas with mean tumor purity 38% (range 6 to 83%)	
Total verified somatic (TP)	717
qSNP pass calls	
*Verified somatic*	704
*Verified germline*	28
*Verified wild type*	506
Precision TP/(TP+FP)	57%
Sensitivity TP/(TP+FN)	98%

### Sequence homology regions

Regions of sequence homology can cause problems in mapping and reads may be erroneously mapped to the wrong homologue. This is not always apparent from the mapping quality values that can remain high especially if these values reflect pairing quality values that consider the mapping qualities of both reads in a read pair. Nevertheless, these regions can often be identified on the basis of having an excess of putative sequence variants. To overcome this challenge, qSNP has a user-defined BAM filtering option so that only high quality reads will trigger a mutation call. For SOLiD v4 data, mapped with Bioscope 2.1 we find the following filters useful:

1. min. 35 bp alignment length or (second of read pair and mapped as a proper pair);
2. min. SM>15 (single mapping quality);
3. no more than 2 base-space mismatches to the reference;
4. not a PCR duplicate (Picard MarkDuplicates).

For Illumina 100 bp paired-end data mapped with BWA, we use the following filters:

1. min. SM>10 (single mapping quality);
2. no more than 3 mismatches to the reference;
3. not a PCR duplicate (Picard MarkDuplicates).

These read filters can be specified in the qSNP configuration file using a domain-specific language (DSL). Once a somatic mutation call has been made, the unfiltered non-duplicate pileup from the normal BAM is checked to see if there is any evidence of the variant. These steps help eliminate many of the false positives associated with this common error source.

### Non-independent reads

Picard MarkDuplicates (http://picard.sourceforge.net.) has become the standard tool for identifying PCR duplicates in next-generation sequencing data. Given that PCR is commonly used to amplify DNA for sequencing, likely PCR duplicates need to be identified so they don't inflate allele counts during mutation calling. To identify duplicates Picard MarkDuplicates uses the start coordinates and orientations of both reads of a read pair. Within a set of duplicate read pairs, the read pair with the highest base qualities is retained with the others marked as PCR duplicates. Picard MarkDuplicates does not consider the sequence of the reads, only the alignment start coordinates and orientations.

**Figure 1:**
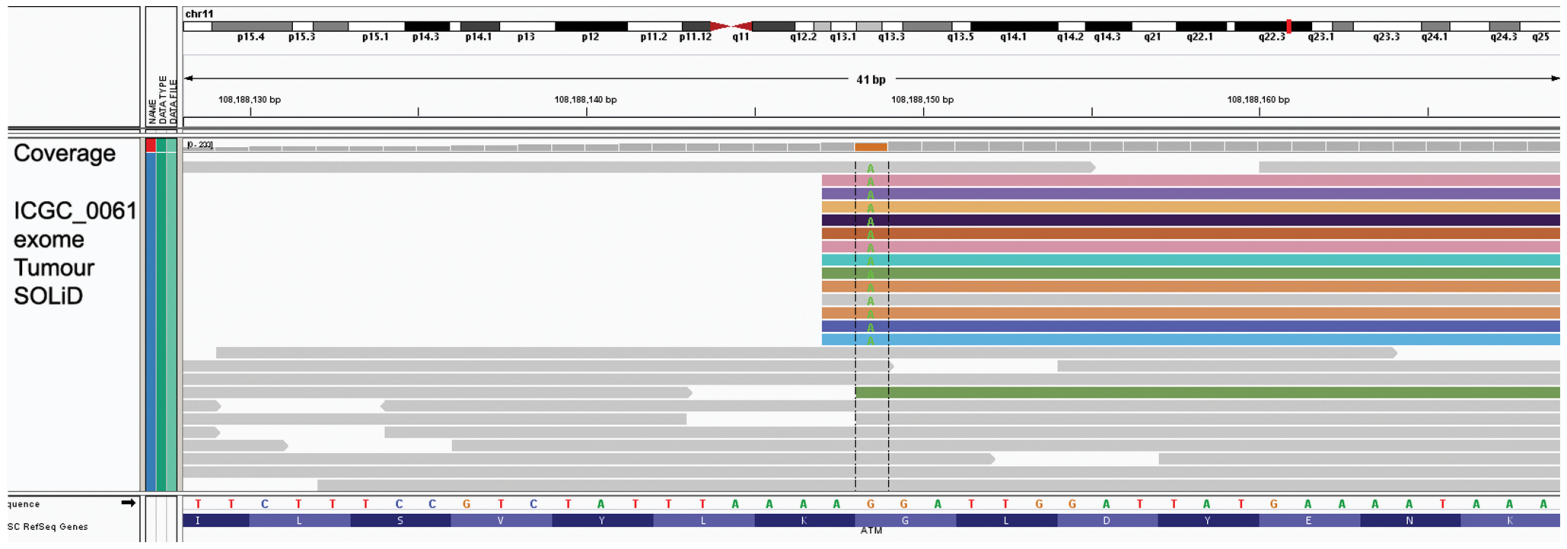
Non-independent reads confounding mutation calls. Read pairs are colored by the chromosome map position of the second read in the pair. MarkDuplicates fails to correctly identify these non-independent read pairs as PCR duplicates due to the different map locations of the second read.

This strategy of marking PCR duplicates has one drawback. Read pairs where one read maps to a region of sequence homology sometimes fail to pass the Picard test for being PCR duplicates because these reads often map to different copies of the region of sequence homology, thus disguising the fact that they are indeed all derived from the same PCR molecule. These reads can be easily identified upon visual inspection in Integrative Genomics Viewer (IGV) [17], [18] on the basis of shared start coordinates of one read partner with different chromosome map positions of the other read in the pair (Figure 1). To overcome this challenge, all putative somatic mutation calls are annotated in qSNP with the number of *novel read starts not considering the read pair* (NNS in the VCF output files). Based on our extensive verification data, we find that a minimum of 4 novel starts using this criterion is a useful lower limit for somatic mutation detection.

### Low evidence calls

Finally, mutation calls that are only supported by a few mutant reads are also common false positives. However, as tumor purity decreases, so does the expected mutant allele ratio, making it difficult to distinguish true somatic events from sequencing artifacts. We investigated a number of criteria to improve signal to noise for calls with low evidence. Strand bias proved not to be a useful discriminating feature for SOLiD v4 data as many true somatic mutations were only supported by reads on one strand. Using results from amplicon-based verification, 363 FP were only on one strand, 171 FP were on both strands, 94 TP were only on one strand, and 610 TP were on both strands. Filtering somatic mutation calls by requiring the mutant allele being represented by reads on both strands will thus severely impact sensitivity of detection. Mutant allele ratio, i.e. proportion of mutant reads, also had poor discriminating power with many true positive calls having very low mutant allele ratios: 112 FP had a mutant allele ratio <10%, 422 FP had a mutant allele ratio >10%, 130 TP had a mutant allele ratio <10% and 574 had a mutant allele ratio >10%.

In contrast, there was a strong positive relationship between the likelihood of being a true somatic event and *the number of reads with novel starts not considering the read pair* supporting the mutation. The higher the number of mutant reads supporting the call, the higher the accuracy. There is a trade-off between sensitivity and accuracy, however. At 5 mutant reads with a minimum of 4 novel starts not considering read pair, we obtain an average accuracy of 57% and sensitivity of 98% (Table 4), which we found useful thresholds for mutation detection and follow-up verification in primary pancreatic adenocarcinomas. By requiring a minimum of 10 mutant reads, our accuracy increases to 94%, but at the cost of reduced sensitivity (53%). These criteria were determined using exome samples that had been sequenced to a depth where 80% of targeted bases had at least 20× coverage (average targeted base coverage of approximately 65×).

### Benchmarking variant calling in a controlled mixture experiment

We previously modeled the performance of qSNP in a panel of mixtures where a pancreatic adenocarcinoma cell line and its matched normal were mixed at the following proportions: 0, 10, 20, 40, 60, 80 and 100% cell line DNA [14]. These mixtures were sequenced to an average depth of approximately 65× using the SureSelect exon capture method and SOLiD v4 sequencing. Here, we compare the decay in sensitivity across these mixtures using variant calls from qSNP and GATK (Table 5). All somatic qSNP calls made in the 100% cell line sample were selected for verification by amplicon-based sequencing on the Ion Torrent PGM. The remaining mutation calls were assessed for evidence on an alternate sequencing platform - HiSeq 2000 for calls made on the SOLiD v4 platform and vice versa. A position was considered verified if read depth was at least 20× and the mutation occurred at a frequency of at least 5% with a minimum of 3 variant reads on the alternate sequencing platform. In all following comparisons, GATK and Strelka were run in default mode with no changes to default parameters. qSNP was run in standard mode, requiring a minimum of 3 mutant alleles of the same type to make a variant call prior to applying standard read annotations and post-calling filters as described in the text.

**Table 5:** Controlled mixture experiment to assess the effect of reducing tumor purity on somatic mutation detection using the SOLiD v4 platform.

Mixture (%tumor)	Cov. 80%	Mean cov.	qSNP			GATK*		
			VS∧	FP∧	U∧	VS∧	FP∧	U∧
100	17×	62.16	84	17	2	50	7	1
80	19×	72.13	73	5	10	49	1	2
60	18×	67.49	66	6	6	45	0	4
40	19×	67.67	57	1	8	38	0	3
20	23×	81.96	35	3	2	15	0	1
10	22×	79.35	13	5	5	0	0	1
20	49×	161.11	48	5	6	18	0	8
10	47×	152.11	15	4	5	0	0	8

* raw.vcf files were passed through qSNP post-processing checks outlined in Table 3 to remove likely false positives such as positions with evidence in the matched normal.

∧ VS verified somatic; FP false positive; U untested.

As expected, as purity decreased, so did the sensitivity of detecting true positive somatic mutations. In total, 84 mutations were verified as true somatic events. At 40% tumor purity, qSNP successfully called 57 of 84 (68%) verified somatic mutations with only 1 false positive call (Table 5). At tumor purities of 20% and 10% the sensitivity of detection dropped to 42% and 15%, respectively. By increasing sequencing depth to >150×, the sensitivity of detection in the 20% and 10% samples was improved, but not to a level comparable to that observed in the higher mixtures (Table 5). In comparison, the GATK pipeline called only 50 of 84 (60%) of verified somatic events in the 100% sample and decayed more rapidly as tumor purity decreased with no successful mutation calls in the 10% mixture (Table 5).

**Figure 2:**
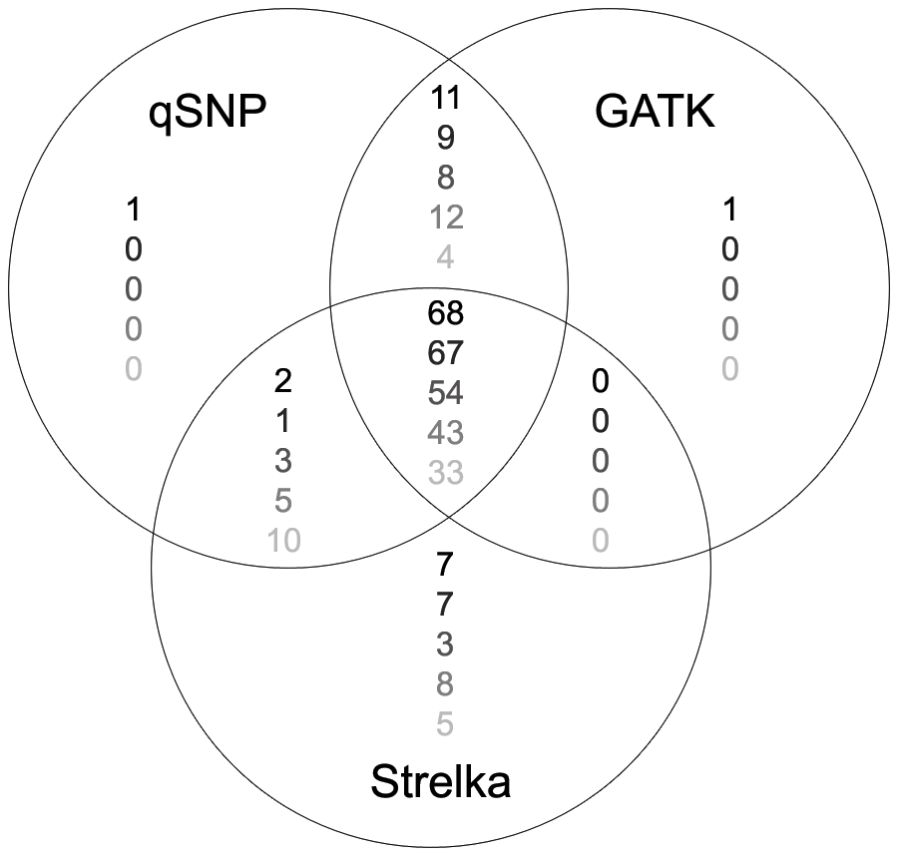
Overlap in somatic mutation calls. Verified somatic mutation calls were compared across three callers in 5 different tumor purity mixtures. Values are number of calls in 100%, 80%, 60%, 40% and 20% tumor content mixture, from top to bottom.

In addition, we re-sequenced 5 of these mixtures to an average depth of 48× on HiSeq 2000 and called mutations using qSNP, GATK and Strelka (Table 6). qSNP detected a greater number of verified somatic events than GATK or Strelka in all mixtures (Table 6). Here, a total of 92 mutations were verified as true somatic events. At 40% tumor purity, qSNP successfully called 60 of 92 (65%) of verified somatic mutations, compared to GATK that called 55 (60%), and Strelka that called 56 (61%) verified somatic mutations (Table 6). There was substantial overlap in true positive somatic calls between the three callers; 68 of a total of 90 (76%) verified somatic mutations were called by all three software tools (Figure 2). These positions had an average of 32 mutant reads with an average mutant allele fraction of 0.52 (range 0.12 to 0.93). As tumor purity decreased, so did the number of mutations called by all three software tools (Figure 2). There were no mutations unique to GATK and Strelka that were not also called by qSNP and for all mixtures qSNP missed the fewest number of true somatic events compared to the other two callers. qSNP and GATK further called 1 private somatic mutation each that was not detected by the other callers, while Strelka called 7 private somatic mutations undetected by the other callers (Figure 2).

**Table 6:** Controlled mixture experiment to assess the effect of reducing tumor purity on somatic mutation detection using the HiSeq2000 platform.

Mixture (%tumor)	Cov. 80%	Mean cov.	qSNP			GATK*			Strelka**		
			VS∧	FP∧	U∧	VS∧	FP∧	U∧	VS∧	FP∧	U∧
100	26×	61.43	82	1	72	80	1	72	77	1	66
80	19×	43.05	77	0	60	76	0	57	75	2	57
60	17×	40.57	65	1	45	62	1	39	60	2	44
40	18×	43.36	60	0	45	55	0	30	56	1	45
20	22×	51.83	47	0	22	37	0	14	48	1	26

* .vcf files were passed through qSNP post-processing checks outlined in Table 3 to remove likely false positives such as positions with evidence in the matched normal.

** calls from ‘pass’ category.

∧ VS verified somatic; FP false positive; U untested.

### COLO-829 whole-genome benchmarking study

The melanoma cell line COLO-829 [4] has been used previously for benchmarking new cancer analysis tools [8], [9]. An aliquot of cell line and matched normal DNA were received and whole-genome sequencing was performed on both SOLiD v4 (avg. coverage 32×) and HiSeq 2000 (avg. coverage 75×). The performance of qSNP was benchmarked against calls previously published by the Wellcome Trust Sanger Institute (WTSI) that included 454 verified somatic mutations, 43 mutations previously reported in COSMIC and 32,842 untested calls [4]. On the SOLiD v4 platform qSNP called 85% of the 454 previously verified somatic mutations as well as 25 novel mutations that were verified using amplicon-based sequencing on the Ion Torrent platform (Table 7, Table S2). For untested calls there was considerable overlap between those reported by Pleasance *et al.* [4] and this study. For all variants called and verified by WTSI but not called by qSNP, a detailed breakdown is provided showing why the call was not made. For example, positions with insufficient coverage in the matched normal and which thus did not pass the qSNP PASS criterion are tabulated as well as positions where we observed evidence in the matched normal sample. The majority of positions where qSNP failed to make a call (5,735 or 62% of positions only called by WTSI) had less than 3 reads evidence in our SOLiD v4 sequence data.

**Table 7:** Benchmarking qSNP on sequencing data from the SOLiD v4 and HiSeq 2000 platforms using COLO-829 variants verified by either WTSI (WTSI only, qSNP+WTSI) or QCMG (qSNP only).

Caller	Details	SOLiD v4			HiSeq 2000			SOLiD v4 and HiSeq 2000		
		VS∧	C∧	U∧	VS∧	C∧	U∧	VS∧	C∧	U∧
**qSNP+WTSI**		381	33	23,544	385	39	23,660	333	30	19,276
**WTSI only**	<12× coverage in normal	18	5	1,329	0	0	104	0	0	26
	mutation also in normal	8	0	455	19	2	1,105	0	0	19
	germline in another patient	0	0	7	1	0	6	0	0	5
	did not pass post-filters	16	1	1,548	24	0	1,623	1	0	86
	qSNP germline call	0	0	24	0	0	63	0	0	10
	no call - <3 reads evidence	0	0	5,735	22	2	5,945	0	0	3,531
	no call - other	31	4	200	3	0	336	2	0	0
**qSNP only** *		25	0	6,486	26	0	13,098	22	0	2,674

* min 5 mutant reads and 4 novel starts not considering pair.

∧ VS verified somatic; C cosmic; U untested.

Using the HiSeq 2000 sequence data, qSNP called 85% of 454 previously reported verified somatic mutations and 26 novel mutations that were verified by Ion Torrent amplicon sequencing (Table 7). Of the positions initially reported by Pleasance *et al.* [4], the two re-sequencing efforts on SOLiD v4 and HiSeq 2000 identified 3531 positions that had less than 3 reads evidence for a mutant allele on both platforms (Table 7). On both platforms qSNP called a significant number of private mutations, 6486 on SOLiD v4 and 13098 on HiSeq 2000 of which 2674 were called on both platforms.

### Germline variants

While qSNP was designed primarily to identify somatic mutations, a comparison of resulting germline calls was made using the COLO-829 sample and calls made by the Illumina Human 1M OmniQuad arrays, selecting all positions from the arrays that showed evidence of a non-reference allele and had a GenCall (GC) score of >0.7. The average genotype concordance at positions with at least 8 reads coverage was 95% (same genotype call), while the variant call concordance was 99% (Table S3). As sequence depth increased so did accuracy in making the correct genotype call. Positions with >70× sequence coverage had a genotype concordance of 99% and a variant call concordance of 100% (Table S3). The array data has been submitted to Gene Expression Omnibus (GEO), accession number GSE47904.

## Discussion

The development of cancer genome analysis tools and somatic mutation calling software is an active area of research, but the effects of reduced tumor purity on somatic mutation calling still remain largely unexplored. Here, we present a strategy for somatic point mutation calling in low purity tumors. We have used extensive verification in primary pancreatic adenocarcinoma samples to determine a variant calling strategy that controls the false positive rate while maximizing sensitivity. When directly assessing the accuracy and sensitivity of our approach in a controlled mixture experiment where samples of varying purity were generated and sequenced, we demonstrate superior performance compared to other commonly used somatic mutation callers, for both SOLiD v4 and HiSeq 2000 data. Finally, we have benchmarked our caller against the COLO-829 sample and show substantial overlap with previously published calls and calls made by either the SOLiD v4 or HiSeq2000 platforms as well as a small number of previously undetected protein-coding somatic mutations.

In the controlled mixture experiment the single sample approach used by GATK had reduced overall sensitivity and a faster decay curve across samples of decreasing tumor purity than the joint sample callers, qSNP and Strelka, consistent with previous reports that joint sample analyses perform better for cancer analysis [9]. Using SOLiD v4 sequence data, qSNP and GATK both achieved a low false positive rate, although GATK called only 60% of known true positives in the 100% purity mixture. Using the HiSeq 2000 platform, the sensitivity of GATK was improved, but at the cost of a high total number of calls likely due to a high false positive rate that was only improved by applying the same post-processing checks as in the qSNP pipeline, such as excluding positions that had evidence of the mutation in the matched normal sample (Table 3).

The controlled mixture experiment further compared our heuristic caller to a Bayesian approach (Strelka), demonstrating a marginal advantage in sensitivity and false positive rate for qSNP. We believe that the success of our heuristic caller is due to its ability to use minimum evidence to trigger a somatic mutation call and the use of powerful post-processing checks that control the false positive rate. Machine learning approaches such as the classifier of Ding *et al.* [6] can be a powerful strategy for identifying features discriminating true positive from false positive mutation calls, provided availability of orthogonal verification data for training of the classifier. Discriminant features can then be incorporated in the set of heuristics for informing mutation calls. In addition, automated pipelines for amplicon-based verification can be set up using smaller scale sequencers such as the Ion Torrent or MiSeq platform. We have found this a successful strategy in pancreatic adenocarcinomas that vary widely in tumor purity. On the other hand, Bayesian approaches may be more readily transferrable across datasets and provide some form of quantitative measure of the confidence for a given mutation call, although as discussed above these will be most useful for high coverage regions and tumors of high purity where allele distributions can be accurately estimated and are not confounded by Poisson sampling effects.

Finally, the controlled mixture experiment demonstrated that no single variant calling strategy is optimal in all aspects. While there was good overlap between callers and the majority of calls were made by at least 2 callers, each caller also identified private mutations not called by the others and which were verified as somatic. Different callers thus have unique benefits, although qSNP missed the fewest number of true somatic events. These comparisons show that there is further scope for refinement of either mutation calling strategy to improve accuracy and sensitivity. Where high-density SNP array data are available, we recommend use of a genomic tool for estimating tumor purity prior to variant calling, such as the qPure software [19]. Determining the purity of a tumor will help identify the most useful thresholds for variant calling. For example, samples of high purity are expected to have a lower false negative rate and thus the stringency of variant calling may be increased to lower the false positive rate. Given that the qSNP analysis of a whole-exome dataset of tumor/matched normal takes only 30 minutes, multiple different parameters can be easily trialled to assess their effect on the total number of calls.

We used the COLO-829 sample for benchmarking both germline and somatic mutation calls. Germline calls from qSNP were compared to those made on the Illumina 1M OmniQuad chip, showing that the variant call concordance was over 99% even for positions with only 8 reads coverage. As sequencing depth increased, so did our accuracy to make the correct genotype call. Detailed comparisons of the qSNP somatic mutation calls against the original GAIIx calls of Pleasance *et al.* [4] showed considerable overlap for re-sequencing data from both the SOLiD v4 and HiSeq 2000 platforms, although there were also some important differences. For example, our re-sequencing efforts identified 3,531 positions that had less than 3 reads evidence for a mutation in both the SOLiD v4 and HiSeq 2000 data, suggesting that these original calls are false positives and may reflect differences in read sampling, mapping or bias of the original sequencing platform. Similarly, calls private to the qSNP pipeline on either the SOLiD v4 or HiSeq 2000 platform likely included a large number of false positive calls as evidenced by the fact that only 2,674of these positions unique to our datasets were called on both sequencing platforms. Our calls on the HiSeq 2000 platform appear noisier judging by the total number of private calls on this platform (13,098) compared to calls on the SOLiD v4 platform (6,486). This is likely due to the increased coverage in the HiSeq runs (75× average base coverage compared to 32× in the SOLiD v4 data), which is expected to result in more variant calls when using the same evidence thresholds. We are currently implementing and refining post-processing checks for use with HiSeq whole-genome datasets that are adjusted for coverage and exclude common error sources, such as calls made in repeat regions, low complexity sequence or near indels. These post-filters are becoming increasingly important as analyses are moving from exon-capture to whole-genome sequencing datasets. Nevertheless, the large overlap in calls between the original and the two re-sequencing datasets suggests that the overall sensitivity of detection of qSNP was good, and that the remaining challenge lies in controlling platform- and software-specific error sources.

## Conclusions

Accurate and sensitive somatic mutation in low purity tumors remains a formidable challenge, but one of great interest to the study of many solid tumors. Here, we have discussed some of the key challenges in this field and strategies we have devised to handle these. Continuous refinement of existing strategies be they heuristics or Bayesian, as well as comparative analyses and benchmarking on a defined set of samples will be critical to further improve performance of current somatic mutation callers.

## Materials and Methods

### Samples

Primary pancreatic adenocarcinoma samples discussed in this study were accrued as part of the Australian Pancreatic Genome Initiative (APGI) (http://www/pancreaticcancer.net.au) using an institutional approved process for consent. COLO-829 sample aliquots for the melanoma cell line and matched normal were obtained from WTSI. Sample extraction and processing followed those outlined in Biankin *et al.* [14].

### Verification of somatic mutations

Verification of somatic mutation calls was performed by targeted Ion Torrent sequencing using PCR primers to amplify 70–150 bp amplicons overlapping the somatic mutation. Tumor and normal DNA was whole-genome amplified prior to PCR using the Illustra GenomiPhi V2 DNA Amplification Kit (GE; 25-6600-30). PCR reactions and sequencing was performed as outlined in Biankin *et al.* (2012). Briefly, PCR reactions were set up using 10 ng of amplified gDNA and 5 uM of primers mix. Ion Spheres were generated using the Ion Xpress Template Kit (Life Technologies; 4469001) with approximately 260 million amplicon molecules per emulsion PCR, effectively yielding an emulsion containing 1 amplicon molecule per Ion Sphere. Samples were sequenced using the Ion Sequencing Kit (Life technologies; 4468997) and the Ion Chip 316 Kit (Life Technologies; 4469496).

Verification of somatic mutations was performed by sequence pileup at each mutant position and a position was considered verified if it has a minimum depth of 100 reads coverage in the tumor and normal, a mutant allele frequency of at least 10% in tumor and less than 0.5% in normal.

### Controlled mixture experiment

SOLiD exon capture data for the mixture experiment was taken from Biankin *et al.* [14].

Illumina exon capture was performed using the TargetSeq Exome Enrichment System (Life Technologies; A14060 and A138230) according to the manufacturer's instructions, however some modifications were made to the protocol to make the kit compatible with Illumina libraries. SOLiD blocking and PCR oligos were replaced with Illumina TruSeq blocking and PCR oligos derived from the NimbleGen SeqCap EZ Library SR User's Guide v3.0 (Roche; 06588786001). The captured libraries were washed on the Life Techologies Library Builder using an unreleased protocol (Life Technologies), and the final post-capture PCR used the protocol in the NimbleGen SeqCap EZ Library SR User's Guide v3.0 (Roche; 06588786001). The final captured libraries were run on the Agilent BioAnalyser 2100 using the DNA High Sensitivity Kit (Agilent; 5067-4626) to calculate the molarity and assess the size distribution. Cluster generation of the libraries was performed using the TruSeq PE Cluster Kit v3-cBot-HS (Illumina; PE-401-3001), and sequencing carried out. The SOLiD and HiSeq.BAM files have been submitted to the European Genome Archive, as part of project EGAS00000000078.

### COLO-829 whole-genome benchmarking study

Whole-genome sequencing of the COLO-829 tumor and matched normal sample were performed using the SOLiD v4 and Illumina HiSeq 2000 sequencing platforms. For preparation of SOLiD v4 long mate-pair libraries, 13 µg of gDNA was sheared to a mean size of 2.5 kb using the Covaris S2 system. Shearing was completed using the Blue miniTUBEs (Covaris p/n: 520065) using the standard settings for 3 kb as described in Covaris protocol 400069 (http://http//covarisinc.com/wp-content/uploads/pn_400069.pdf). Following shearing, 1 uL of sheared sample was run on the Agilent BioAnalyser2100 using the DNA High Sensitivity Kit (Agilent p/n: 5067-4626) to assess the shearing size and distribution. The entire sheared DNA sample was then converted into a SOLiD® compatible Long Mate Pair (LMP) library using Life Technologies 5500SOLiD® Mate-Paired Library Kit (Invitrogen p/n: 4464418) following the standard protocol (http://tools.invitrogen.com/content/sfs/manuals/cms_093442.pdf) with 10 minutes nick translation and a total of 12 cycles of amplification for the final library. After PCR amplification the libraries were assessed for molarity and size distribution using the Agilent BioAnalyser 2100 using the DNA High Sensitivity Kit. Libraries that passed this QC were prepared for SOLiD® sequencing.

For the preparation of Illumina DNA libraries, 1 µg of gDNA was sheared to a mean size of 300 bp in a 130 µL volume using a Covaris microTUBE and the Covaris S2 system according to the standard protocol (Covaris; 010158 Rev C). The sheared sample was prepared into a library using the NEBNext DNA Library Prep Master Mix Set for Illumina (NEB; E6040S) according to the manufacturer's instructions with modifications. Size selection was done using an agarose gel (3% agarose) instead of the AMPure XP Beads size selection. The final libraries were run on the Agilent BioAnalyser 2100 using the DNA High Sensitivity Kit (Agilent; 5067-4626) to calculate the molarity and assess the size distribution. Libraries were then prepared for Illumina cluster generation and sequencing.

Of the qSNP unique calls, 61 protein-coding positions were selected for verification on the Ion Torrent platform using the same verification criteria as outlined above; 30 were confirmed as true somatic events and 31 as false positives (Table S2). In addition, 3 somatic mutations originally identified by WTSI could not be confirmed as somatic events in our verification efforts (Table S2).

## Supporting Information

Table S1 **Verification of 3253 putative somatic mutation calls across 65 tumors.**
(XLSX)

Table S2 **A mutation file containing somatic mutations for the COLO-829 data set.**
(XLSX)

Table S3 **Comparison of qSNP germline variant calls to calls from SNP array analysis.**
(DOCX)
