# Supplementary material for: Somatic Point Mutation Calling in Low Cellularity Tumors
Source: PLoS One. 2013 Nov 8;8(11):e74380. doi: 10.1371/journal.pone.0074380 (PMC3826759; doi:10.1371/journal.pone.0074380)
Supplement: Table S3 — Comparison of qSNP germline variant calls to calls from SNP array analysis. (DOCX) [file pone.0074380.s003.docx]

**Table S3. Comparison of qSNP germline variant calls to Illumina 1M OmniQuad array calls.**

| **Coverage** | **number of SNPs** | **Genotype concordance** | **% genotype concordance** | **Variant call concordance** | **% variant call concordance** |
| --- | --- | --- | --- | --- | --- |
| 0 | 6,147 | n/a | n/a | n/a | n/a |
| 1-7 | 8,612 | 6,021 | 69.91 | 6,716 | 77.98 |
| 8-19 | 70,690 | 64,509 | 91.26 | 68,740 | 97.24 |
| 20-29 | 125,341 | 120,073 | 95.80 | 124,787 | 99.56 |
| 30-39 | 108,643 | 104,855 | 96.51 | 108,589 | 99.95 |
| 40-49 | 41,142 | 39,713 | 96.53 | 41,134 | 99.98 |
| 50-59 | 8,074 | 7,936 | 98.29 | 8,073 | 99.99 |
| 60-69 | 1,017 | 1,005 | 98.82 | 1,017 | 100.00 |
| >70 | 104 | 103 | 99.04 | 104 | 100.00 |
| **>8** | **355,011** | **338,194** | **95.26** | **352,444** | **99.28** |
